# Autologous stem cell transplantation in NK/T‐cell lymphoma: Prognostic impact of EBV‐DNA in a multinational cohort—A study by the EBMT Lymphoma Working Party

**DOI:** 10.1002/hem3.70184

**Published:** 2025-08-15

**Authors:** Philipp Berning, Maud Ngoya, Won Seog Kim, Evgenii Shumilov, Depei Wu, Haiwen Huang, Anne Cairoli, Alessandra Tucci, Guillaume Dachy, Romain Gounot, Anne C. Wilke, Christof Scheid, Peter Dreger, Jose Luis Lopez Lorenzo, Adrian Bloor, Joanna Romejko‐Jarosinska, Alain Gadisseur, Roland Schroers, Péter Reményi, Ludovic Gabellier, Monica Poiani, Khalid Halahleh, Jacques‐Emmanuel Galimard, Georg Lenz, Anna Sureda, Ali Bazarbachi, Bertram Glass, Norbert Schmitz

**Affiliations:** ^1^ Department of Medicine A, Hematology, Oncology and Pneumology University Hospital Muenster Muenster Germany; ^2^ European Society for Blood and Marrow Transplantation Paris France; ^3^ Sungkyunkwan University School of Medicine, Samsung Medical Center Seoul South Korea; ^4^ National Clinical Research Center for Hematologic Diseases, Jiangsu Institute of Hematology The First Affiliated Hospital of Soochow University Suzhou China; ^5^ Division of Haematology, Department of Oncology University Hospital of Lausanne (CHUV) Lausanne Switzerland; ^6^ Department of Hematology ASST Spedali Civili Brescia Italy; ^7^ Hematology Department Cliniques Universitaires Saint‐Luc Brussels Belgium; ^8^ Service Hématologie Hôpital Henri Mondor Creteil France; ^9^ University Hospital Frankfurt, Goethe University Frankfurt Main Germany; ^10^ Department I of Internal Medicine, Medical Faculty and University Hospital of Cologne University of Cologne Cologne Germany; ^11^ Department of Medicine V University of Heidelberg Heidelberg Germany; ^12^ Hematology Department Hospital Universitario Fundación Jiménez Díaz UAM Madrid Spain; ^13^ Christie Hospital NHS Foundation Trust Manchester UK; ^14^ Department of Lymphoid Malignancies Maria Sklodowska‐Curie National Research Institute of Oncology Warsaw Poland; ^15^ Department of Hematology University Medical Center Antwerpen Edegem Belgium; ^16^ Medizinische Klinik II, Knappschaft Kliniken Universitätsklinikum Bochum Bochum Germany; ^17^ Dél‐pesti Centrumkórház‐Országos Hematológiai és Infektológiai Intézet, Dept. Haematology and Stem Cell Transplant Budapest Hungary; ^18^ Hematology Department University Hospital of Montpellier Montpellier France; ^19^ UCO Ematologia, Azienda Sanitaria Universitaria Giuliano Isontina Trieste Italy; ^20^ King Hussein Cancer Centre Adult BMT Program Amman Jordan; ^21^ Hematology Department Institut Català d'Oncologia Hospitalet, IDIBELL, Universitat de Barcelona Barcelona Spain; ^22^ Department of Internal Medicine American University of Beirut Medical Center, Hematology‐Oncology Division Beirut Lebanon; ^23^ Department of Hematology and Stem Cell Transplantation Helios Clinic Berlin‐Buch Germany

## Abstract

Natural killer (NK)/T‐cell lymphomas (NKTCLs) are rare, aggressive lymphomas prevalent in East Asia and South America. Despite improvements, largely due to asparaginase‐based therapies, outcomes for advanced disease remain poor, and the role of autologous stem cell transplantation (auto‐HCT) remains controversial. This study evaluated real‐world outcomes of auto‐HCT in NKTCL patients across Asia and Europe. We included 130 adult NKTCL patients undergoing auto‐HCT between 2011 and 2022 using data from the European Society for Blood and Marrow Transplantation (EBMT) registry and South Korean registry data. Median age was 51.3 years; 66.9% were male. Most patients (95.1%) had an Eastern Cooperative Oncology Group (ECOG) score 0–1; 65.3% had Stage III–IV disease. One prior therapy line was reported in 53.1%, and ≥2 lines in 46.9%. Asparaginase‐based regimens were used in 79.5% pretransplant. Responses at auto‐HCT included complete (59.7%), partial (27.9%) remission, and stable/progressive disease (12.4%). Epstein–Barr virus (EBV)‐DNA in the peripheral blood was reported in 37.3%. With a median follow‐up of 4.6 years, 3‐year overall survival (OS) and progression‐free survival (PFS) were 63.8% and 47.6%. Relapse and NRM rates at 3 years were 46.7% and 5.7%. Patients in complete remission had improved 3‐year OS (75.2%) compared to PR (52.8%) and stable/progressive disease (32.0%) (P = 0.007). Detectable EBV‐DNA in the blood at auto‐HCT was associated with poor outcomes (3‐year OS: 26.7% vs. 78.1% in patients with undetectable EBV‐DNA; P < 0.0001). Patients achieving complete remission and undetectable EBV‐DNA in the blood before auto‐HCT had a favorable survival, suggesting auto‐HCT may be a treatment option in selected high‐risk patients. This is the largest multinational cohort evaluating prognostic factors for auto‐HCT for NKTCL.

## Graphical abstract

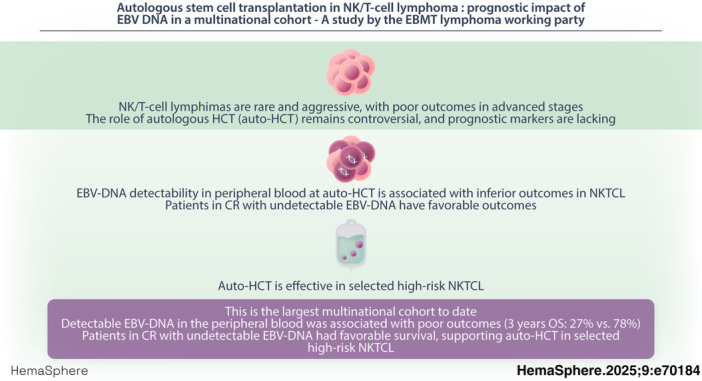

## INTRODUCTION

Natural killer (NK)/T‐cell lymphomas (NKTCLs) represent a rare and aggressive group of lymphoid malignancies of NK‐cell or cytotoxic T‐cell origin.^1^, ^2^ While extranodal NKTCL is a well‐defined entity according to the WHO and ICC classifications, less common, primary nodal forms of NKTCLs also do occur.^1^, ^2^, ^3^ Both presentations are often referred to as NKTCL, reflecting shared clinical features and therapeutic considerations in clinical routine. Their prevalence shows regional differences, representing more than 5%–10% of lymphomas in East Asia and South America being notably less common in Western populations.^4^, ^5^, ^6^ A hallmark of NKTCLs is the presence of Epstein–Barr virus (EBV), with peripheral viral DNA load serving as a biomarker for assessing disease activity and prognosis.^7^, ^8^ Current therapeutic approaches, particularly those utilizing asparaginase (ASPA), have significantly improved treatment outcomes.^9^, ^10^, ^11^, ^12^ Nevertheless, an unmet need remains to further improve treatment, especially for patients with advanced‐stage or relapsed/refractory (r/r) disease.

Patients are typically managed with ASPA‐based regimens, showing overall survival (OS) rates of 40%–50%.^9^, ^10^, ^11^ Current NCCN and ESMO/EHA guidelines suggest consolidative autologous stem cell transplantation (auto‐HCT) or allogeneic stem cell transplantation (allo‐HCT) for patients in remission following first‐line therapy.^13^, ^14^ However, the evidence to support consolidative transplantation and how to choose between auto‐ and allo‐HCT is limited to retrospective analyses mostly including small numbers of patients. Although allo‐HCT is a well‐known viable option for high‐risk patients,^15^ its use for patients in remission after first‐line therapy is hampered by concerns regarding treatment‐related morbidity and mortality.^15^, ^16^ The role of auto‐HCT, either to consolidate remission upfront or to treat patients with r/r disease, is poorly described, not allowing firm conclusions on how to integrate auto‐HCT into current treatment algorithms. Recent reports on the use of immune checkpoint inhibitors (ICIs) targeting the PD‐1/PD‐L1 pathway have shown promise in r/r NKTCL,^17^, ^18^, ^19^ with emerging evidence supporting their use also in first‐line treatment.^20^, ^21^

This European Society for Blood and Marrow Transplantation (EBMT) registry‐based study evaluates the outcomes of NKTCL patients treated with auto‐HCT following state‐of‐the‐art therapy, which in some cases included ICI. These findings offer insights into the role of auto‐HCT within a contemporary real‐world context, providing improved guidance on how to integrate auto‐HCT into the current treatment algorithms for this challenging lymphoma.

## METHODS

### Data collection

We performed a retrospective analysis of transplant data registered with the Lymphoma Working Party of the EBMT, supplemented by the local registry from the Samsung Medical Center, Seoul, South Korea. In total, data were provided by 43 transplant centers across Europe and Asia (Table S1 lists all centers and patient numbers). Further details regarding the standardized data collection and quality management processes can be found elsewhere.^22^ The present study has been approved by the EBMT Lymphoma Working Party, and all accredited EBMT centers obtain informed consent before data registration with EBMT, in accordance with the Helsinki Declaration of 1975. The cooperating center signed a project‐specific joint controllership agreement, confirming adherence to all EBMT regulations as well as the requirements of local institutional review boards or ethics committees, in accordance with regional guidelines.

We included data for consecutive adult patients (≥18 years) diagnosed with NKTCL, regardless of prior treatment and remission status, who received auto‐HCT as first transplantation. Data of transplantations performed between 2011 and 2022 were analyzed to capture patients treated with state‐of‐the‐art conventional therapy and to ensure adequate follow‐up. Baseline characteristics, transplantation characteristics as well as outcome data were extracted from the EBMT registry. Identical reporting forms were provided by the center not being EBMT member.

### Definitions

Histological diagnosis of NKTCL was based on local pathological review. Disease type was reported as either “nasal‐type” or as having “extranasal manifestations,” the latter including nodal presentations. Cases with incomplete data were classified as unknown. Disease stages were classified according to the Ann Arbor system as localized (Stage I/II) and advanced (Stage III/IV); the prognostic index for NK/T‐cell lymphoma (PINK) scoring system was applied to classify patients belonging to the different risk groups as published previously.^8^ In 34 patients with PINK scores not reported, we constructed scores for 25 patients based on the available clinical data.^8^ Due to heterogeneous methodologies and center‐specific variations in the quantitative detection of EBV‐DNA in the peripheral blood via polymerase chain reaction in either patient plasma or whole blood, quantitative analyses to determine cutoff values were not feasible. For the present analysis, any reported detectable concentration/copy number indicating detectable EBV‐DNA was defined as positive and was included in the analyses.

Relapse was diagnosed when lymphoma recurred at least 3 months after the end of all therapies in patients having achieved a complete remission (CR). Allo‐HCT following failure of auto‐HCT was considered as a relapse event, with allo‐HCT considered a relapse treatment. Disease status was assessed by individual investigators according to standard criteria at the time patients were referred for transplantation. Conventional chemotherapeutic regimens applied before auto‐HCT were categorized as ASPA‐based, anthracycline‐based, or platinum‐based.

### Statistical analysis

The endpoints analyzed were progression‐free survival (PFS) defined as survival without lymphoma relapse or progression (patients alive without lymphoma relapse or progression were censored at the time of last contact), OS defined as time from transplantation to death from any cause; non‐relapse mortality (NRM) defined as death without previous lymphoma relapse and relapse incidence (RI). All outcomes were measured from the day of transplantation. Surviving patients were censored at the time of last contact. The probabilities of OS and PFS were calculated using the Kaplan–Meier method. We calculated cumulative incidences for RI and NRM using a competing risk model, where death during remission was treated as a competing event for relapse. Demographics were compared between groups using the chi‐squared test or Fisher's exact test for categorical variables. Except for OS, where all 130 patients were analyzed, outcomes including RI and PFS, were analyzed in 124 patients. Six patients had to be excluded from these analyses due to incomplete information.

Univariable analyses were performed using the log‐rank test for PFS and OS, whereas Gray's test was used for cumulative incidences. Multivariable analyses were performed using the Cox proportional‐hazards regression model. Results were shown as hazard ratio (HR) with 95% confidence intervals (95% CIs). All tests were two‐sided, and the Type I error was fixed at 0.05 for factors associated with time to event outcomes. All analyses were performed using R statistical software version 4.4.3 (available online at http://www.R-project.org) and IBM SPSS version 30.0 (SPSS Inc., Chicago, IL).

## RESULTS

### Patient characteristics, pretreatment, and transplant modalities

The study included 130 patients receiving auto‐HCT between 2011 and 2022. Major patient‐ and transplant‐related characteristics are shown in Table 1. Our cohort is representative for a typical NKTCL population^23^ with a median age of 51.3 years (range: 23.0–72.8 years) at transplantation and 66.9% of patients were male. Eastern Cooperative Oncology Group (ECOG) performance scores of 0–1 at auto‐HCT were reported for most of the patients (95.1%). Nasal‐type NKTCL was present at diagnosis in 53.9% of the cases. Advanced disease status at diagnosis (Stages III–IV) was noted in 65.3%; high PINK scores were reported for 31.4% of the evaluable cases. First‐line therapies are summarized in Table 1; most of the patients (65.4%) received ASPA as part of first‐line treatment, 12.3% had an anthracycline‐based regimen, 8.5% were treated with platinum‐based regimens (including dexamethasone, etoposide, ifosfamide, carboplatin [DeVIC], etoposide, ifosfamide, cisplatin, dexamethasone [VIPD], or ifosfamide, carboplatin, etoposide [ICE]), and 10.8% had radiotherapy only. Detailed information on radiotherapy, including dose and field, was not consistently available in the EBMT registry and could not be evaluated. Last treatment regimens before autologous transplantation are listed in Table 1; ASPA‐based regimens were given to most patients (66.3%), followed by anthracycline‐based regimens (13.5%). Approximately half of the patients (53.1%) received auto‐HCT after one prior treatment line (upfront), whereas 46.9% had two or more prior lines of therapy. PD‐1/PD‐L1 inhibitors were administered to 17 patients (13.6%) before auto‐HCT. Overall, before auto‐HCT, 79.5% of the patients had received ASPA‐containing regimens. Remission status at the time of HCT was CR in 59.7%, partial remission (PR) in 27.9%, and stable/progressive disease (SD/PD) in 12.4%. Among CR patients, 65.3% had achieved CR after 1 line of treatment (CR1), whereas 34.7% achieved CR after 2 or more treatment lines (CR2) before auto‐HCT. EBV‐DNA at auto‐HCT was reported in 83 of 130 patients (63.8%), of which 37.3% had detectable EBV‐DNA levels at transplantation. Remission status at auto‐HCT by disease stage and number of prior treatment lines is summarized in Table S2. Notably, most patients with Stage I/II disease had received 2 or more lines of therapy (66.7%) and/or underwent HCT while not in CR (38.1%), thus comprising a high‐risk population.

**Table 1:** Clinical and transplantation characteristics for all natural killer/T‐cell lymphoma patients.

Variable	Total cohort *N* = 130 (%)
Age at transplantation, median, range (years)	51.3 (23.0–72.8)
Diagnosis—auto‐HCT median, range (months)	7.51 (2.8–174.9)
Male	87 (66.9)
ECOG at auto‐HCT	
0–1	58 (95.1)
≥2	3 (4.9)
Unknown	69
Region	
Asia	56 (43.1)
Europe	74 (56.9)
Disease type	
Extranasal manifestations	53 (46.1)
Nasal‐type	62 (53.9)
Unknown	15
Ann‐Arbor stage at diagnosis	
Localized (I–II)	42 (34.8)
Advanced (III–IV)	79 (65.3)
Unknown	9
PINK score	
High	38 (31.4)
Low/intermediate	83 (68.6)
Unknown	9
First‐line therapy	
Asparaginase‐based	85 (65.4)
Anthracycline‐based	16 (12.3)
Radiotherapy only	14 (10.8)
Platinum‐based (DeVIC/VIPD/ICE)	11 (8.5)
Other	4 (3.1)
Last treatment before auto‐HCT	
Asparaginase‐based	69 (66.3)
Platinum‐based (DeVIC/VIPD/ICE)	8 (7.7)
Anthracycline‐based	14 (13.5)
Other	12 (11.5)
Radiotherapy only	1 (1.0)
Unknown	26
Number of prior therapies	
1	68 (53.1)
2	40 (31.3)
3 or more	20 (15.6)
Unknown	2
Asparaginase‐containing therapy (any time before HCT)	97 (79.5)
PD‐1/PD‐L1 inhibitor treatment	
No PD‐1/PD‐L1 inhibitor	108 (86.4)
Before HCT	17 (13.6)
Unknown	5
Status at transplantation	
CR	77 (59.7)
CR1 (after 1 line of treatment)	49 (65.3)
CR2 (after 2 or more lines of treatment)	26 (34.7)
CR unknown number of treatment lines	2
PR	36 (27.9)
SD/PD	16 (12.4)
Unknown	1
EBV‐DNA at transplantation	
Detectable	31 (23.8)
Not detectable	52 (40.0)
Not evaluated/unknown	47 (36.2)
Conditioning regimen	
BEAM	46 (39.0)
BEAM‐like	33 (28.0)
TBI based	37 (31.4)
TBI + BEAM/BEAM‐like	2 (1.7)
Unknown	12
Subsequent allo‐HCT	13 (10.0)

Abbreviations: allo‐HCT, allogeneic hematopoietic stem cell transplantation; auto‐HCT, autologous hematopoietic stem cell transplantation; BEAM, carmustine, etoposide, cytarabine, melphalan; CR, complete remission; DeVIC, dexamethasone, etoposide, ifosfamide, carboplatin; EBV, Epstein–Barr virus; ECOG, Eastern cooperative oncology group; ICE, ifosfamide, carboplatin, etoposide; PD, progressive disease; PINK, prognostic index for NK/T‐cell lymphoma; PR, partial remission; SD, stable disease; TBI, total body irradiation; VIPD, etoposide, ifosfamide, cisplatin, dexamethasone.

Regarding the preparatory regimens used, carmustine, etoposide, cytarabine, melphalan (BEAM; 39.0%) and BEAM‐like regimens (28.0%) were the most frequently used, followed by total body irradiation (TBI)‐based conditioning (31.4%). TBI as part of high‐dose therapy was applied in 33.1% of all cases with TBI being strikingly more frequent among Asian compared to European patients (71.1% vs. 9.5%, P < 0.0001; Table S3). Thirteen (10.0%) patients of this cohort underwent a subsequent allo‐HCT for relapse/progression.

When comparing patient characteristics between Asian and European patients, significant differences in key parameters were observed (Table S3). While median age at transplantation and gender distribution were similar, there were marked differences in the frequencies of high PINK score group (12.5% in Asian vs. 47.7% in European patients, P < 0.001), three or more prior lines of treatment (25% vs. 8.3%, P = 0.021), and the use of subsequent allo‐HCT (4.5% vs. 20%, P = 0.023) (Table S3). Regarding the disease status at the time of auto‐HCT, Asian patients were less frequently in CR (49.1% vs. 67.6%), but more commonly in PR (34.5% vs. 23%) compared to European patients (Table S3).

### Transplantation outcomes

**Figure 1:**
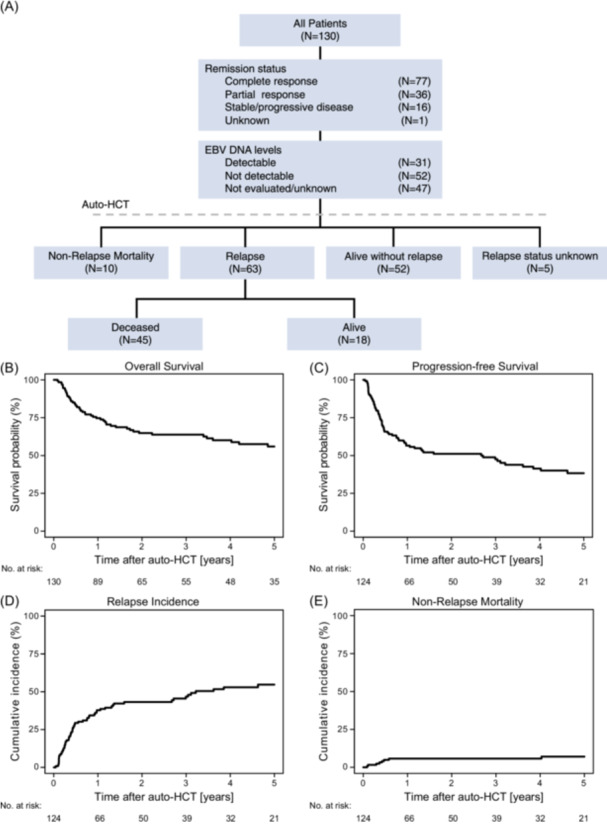
**Patient flow and major outcomes of natural killer/T‐cell lymphoma (NKTCL) patients after autologous stem cell transplantation (auto‐HCT).** Patient flow and key outcome parameters for NKTCL patients undergoing auto‐HCT. **(A)** Flow diagram for patterns of treatment and outcomes. Kaplan–Meier estimates for **(B)** overall survival (OS) and **(C)** progression‐free survival (PFS). Cumulative incidence curves for **(D)** relapse incidence (RI) and **(E)** non‐relapse mortality (NRM). Time‐to‐event data for PFS, RI, and NRM were available for 124 patients, as shown in panels **(C)–(E)**. EBV, Epstein‐Barr virus.

Major outcomes of patients receiving auto‐HCT are shown in Figure 1 and Table 2. Figure 1A shows a flowchart summarizing the treatment outcomes and patient characteristics, including remission status before transplantation, EBV‐DNA detectability at the time of auto‐HCT, and posttransplantation outcomes. With a median follow‐up of 4.6 years (95% CI: 4.1–6.0 years), OS and PFS were 74.7% (95% CI: 66.1–81.4) and 56.5% (95% CI: 47.3–64.8) at 1 year, and 63.8% (95% CI: 54.4–71.7) and 47.6% (95% CI: 38.1–56.4) at 3 years, respectively (Figure 1B,C). The corresponding RI was 37.7% (95% CI: 29.1–46.3) and 46.7% (95% CI: 37.2–55.6) at 1 and 3 years, respectively; NRM reached 5.7% (95% CI: 2.5–10.8) at 1 and 3 years (Figure 1D,E). Fifty‐five patients had deceased at the last follow‐up. Disease relapse was the most common cause of death, occurring in 37 patients (69.8%), followed by complications related to HCT in 7 patients (13.2%). Other causes of death were reported in 8 cases (15.1%), which included 3 patients with infectious complications, 1 patient (1.9%) who developed a secondary malignancy (myelodysplastic syndrome), and 5 patients with unknown causes of death.

**Table 2:** Posttransplantation outcomes for all natural killer/T‐cell lymphoma patients.

Outcomes	*N*	Probability (95% CI)
Relapse incidence	124	
1 year		37.7% (29.1−46.3)
3 years		46.7% (37.2−55.6)
5 years		54.7% (44.2−63.9)
Non‐relapse mortality	124	
1 year		5.7% (2.5−10.8)
3 years		5.7% (2.5−10.8)
5 years		7.0% (3.2−12.8)
Progression‐free survival	124	
1 year		56.5% (47.3−64.8)
3 years		47.6% (38.1−56.4)
5 years		38.3% (28.7−47.9)
Overall survival	130	
1 year		74.7% (66.1−81.4)
3 years		63.8% (54.4−71.7)
5 years		55.9% (45.8−64.9)

Abbreviation: CI, confidence interval.

### Outcomes across subgroups

**Figure 2:**
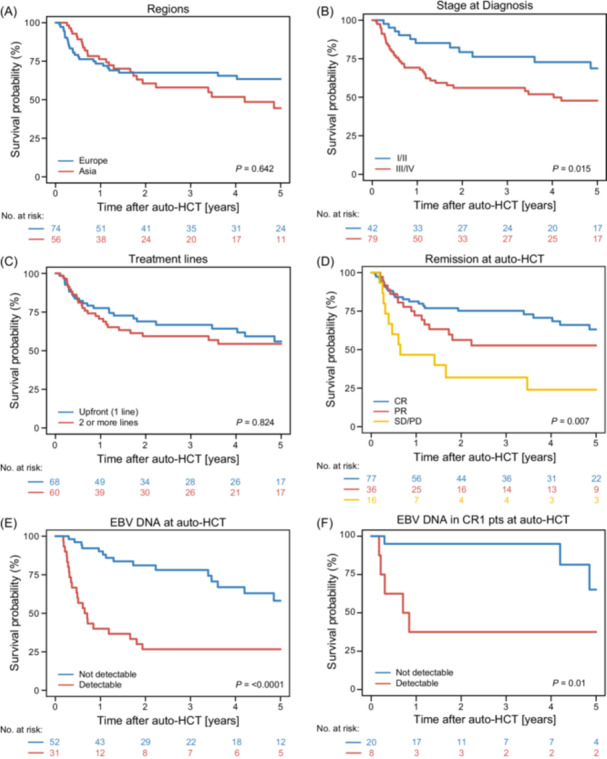
**Overall survival of natural killer/T‐cell lymphoma patients by region and disease factors.** Kaplan–Meier estimates for overall survival of indicated subgroups. **(A)** Patients transplanted in European or Asian centers. **(B)** Limited disease stage (I/II) versus advanced disease stage (III/IV) at diagnosis. **(C)** Timing of autologous stem cell transplantation (auto‐HCT) as upfront treatment (after 1 treatment line) versus 2 or more treatment lines. **(D)** Remission status at autologous stem cell transplantation (auto‐HCT) shown as CR versus PR versus SD/PD. **(E)** Epstein–Barr virus (EBV)‐DNA not detectable versus detectable at auto‐HCT. **(F)** CR1 patients stratified by EBV‐DNA not detectable versus detectable at auto‐HCT. CR, complete remission; CR1, complete remission after 1 treatment line; PR, partial remission; SD/PD, stable disease/progressive disease.

Three‐year OS rates were comparable between Asian and European patients (60.6% [95% CI: 45.4–72.7] vs. 67.6% [95% CI: 55.3–77.1]; P = 0.642), RI also did not differ significantly, although numerically higher relapse rates were observed in Asian patients (Table 3, Figure 2A). Patients with localized disease (Stage I/II) had significantly better survival compared to those with advanced‐stage disease (III/IV), with a 3‐year OS of 76.2% (95% CI: 59.1–86.9) versus 56.1% (95% CI: 44.0–66.6; P = 0.015) and corresponding PFS of 61.0% versus 41.7% (P = 0.040) (Table 3, Figure 2B).

**Table 3:** Univariate analysis of posttransplantation outcomes for all patients.

Variable		3‐Year probability [95% CI]		
Region	Outcome	Asia	Europe	P
	OS	60.6% [45.4–72.7]	67.6% [55.3–77.1]	0.642
	PFS	45.9% [31.7–59.1]	54.9% [42.6–65.6]	0.238
	RI	48.2% [33.5–61.4]	39.5% [28–50.7]	0.141
	NRM	5.9% [1.5–14.8]	5.6% [1.8–12.6]	0.551

**Disease stage at diagnosis**		**I/II**	**III/IV**	
	OS	76.2% [59.1–86.9]	56.1% [44–66.6]	0.015
	PFS	61% [43.6–74.4]	41.7% [29.9–53.1]	0.040
	RI	36.5% [21.4–51.8]	50.2% [37.7–61.5]	0.171
	NRM	2.5% [0.2–11.4]	8.1% [3.3–15.7]	0.258

**Treatment lines before auto‐HCT**		**Upfront (1 line)**	**2 or more lines**	
	OS	66.7% [53.3–77]	59.4% [45.3–70.9]	0.824
	PFS	47.9% [34.6–60.1]	45.5% [31.9–58.1]	0.586
	RI	47.4% [33.9–59.7]	47.4% [33.5–60.1]	0.563
	NRM	4.6% [1.2–11.8]	7.1% [2.3–15.9]	0.976

**Remission status at auto‐HCT**		**CR**	**Non‐CR**	
	OS	75.2% [63.5–83.7]	46.5% [31.9–60]	0.068
	PFS	55.3% [42.3–66.6]	37.7% [24.5–50.9]	0.185
	RI	40.5% [28.2–52.4]	54.3% [39.2–67.1]	0.192
	NRM	4.2% [1.1–10.7]	8% [2.5–17.7]	0.902

**EBV‐DNA at auto‐HCT**		**Detectable**	**Not detectable**	
	OS	26.7% [12.6–43]	78.1% [62.7–87.7]	<0.0001
	PFS	26.7% [12.6–43]	52.2% [35.6–66.4]	0.002
	RI	66.7% [46.1–80.9]	41.7% [25.9–56.8]	0.007
	NRM	6.7% [1.1–19.8]	6.1% [1.6–15.3]	0.684

Abbreviations: auto‐HCT, autologous hematopoietic stem cell transplantation; CI, confidence interval; CR, complete remission; EBV, Epstein–Barr virus; NRM, cumulative incidence of non‐relapse mortality; OS, overall survival; PFS, progression‐free survival; RI, cumulative incidence of relapse.

No significant differences in OS or PFS were observed between nasal‐type and extranasal cases. This finding also applied when PINK risk groups were considered (Table S4, Figure S1A). The timing of auto‐HCT (upfront vs. after ≥2 lines) showed no significant impact on outcomes (Figure 2C). The 3‐year OS rates were 66.7% (95% CI: 53.3%–77.0%) and 59.4% (95% CI: 45.3%–70.9%) (P = 0.824), respectively. These findings remained consistent after excluding patients who had received anthracycline‐based therapy (Table S4). Similarly, no significant differences were observed between patients receiving TBI‐based versus BEAM‐based preparatory regimens (Table S4).

Among patients with advanced‐stage disease at diagnosis, those receiving upfront auto‐HCT had numerically higher 3‐year OS (64.1% [95% CI: 49.1–75.8]) compared to those transplanted after ≥2 lines (40.6% [95% CI: 21.6–58.8]; P = 0.210), although this did not reach statistical significance (Table S4, Figure S1B).

Remission status at transplant was strongly associated with survival. OS was significantly lower in patients transplanted in SD/PD (32.0%, 95% CI: 10.9–55.7) compared to those in CR (75.2%, 95% CI: 63.5–83.7) or PR (52.8%, 95% CI: 34.5–68.1; P = 0.007) (Figure 2D). No significant difference was observed between CR1 and CR2 patients, with 3‐year OS rates of 76.9% (95% CI: 62.1–86.5) and 71.4% (95% CI: 49.1–85.2), respectively (P = 0.94; Figure S1C).

The presence of EBV‐DNA in peripheral blood at the time of auto‐HCT was associated with worse outcomes: 3‐year OS was 26.7% (95% CI: 12.6–43.0) compared to 78.1% (95% CI: 62.7–87.7; P < 0.0001) in patients with undetectable EBV‐DNA. The decrease in OS was mainly driven by a significantly higher RI (P = 0.007) (Table 3, Figure 2E). Also, among patients transplanted in CR1, those with detectable EBV‐DNA had markedly lower OS (37.5%, 95% CI: 8.7–67.4) compared to patients with undatable EBV‐DNA (95.0%, 95% CI: 69.5–99.3; P = 0.01) (Figure 2F). Of note, prior PD(L)−1‐directed therapy had no significant impact on survival outcomes (Table S4).

### Multivariable regression analysis

**Figure 3:**
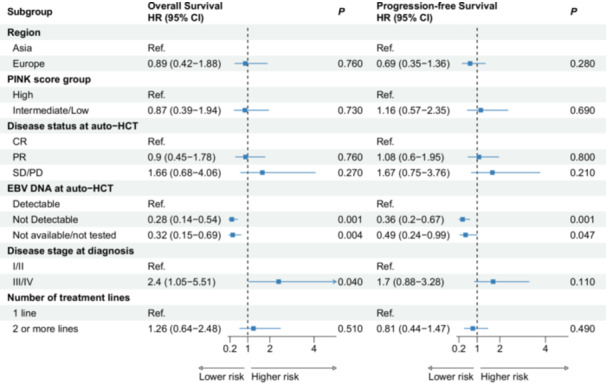
**Forest plots of multivariable analysis of prognostic factors for OS and PFS.** The hazard ratios (HR) and corresponding 95% confidence intervals (95% CIs) were obtained from multivariable regression using the Cox proportional‐hazards model. These results pertain to the independent variables evaluated for predicting post‐autologous stem cell transplantation (auto‐HCT) outcomes in all natural killer/T‐cell lymphoma patients. CR, complete remission; EBV, Epstein‐Barr virus; OS, overall survival; PFS, progression‐free survival; PINK, prognostic index for NK/T‐cell lymphoma; PR, partial remission; Ref., reference group; SD/PD, stable disease/progressive disease.

To evaluate the prognostic impact of disease stage, disease status, and timing of auto‐HCT, we used a multivariable regression model that included region, PINK score categories, disease status at auto‐HCT, EBV‐DNA viral load at auto‐HCT, disease stage at diagnosis, and the number of prior treatment lines (Figure 3). Univariable analysis showed lower PFS and OS rates in patients with advanced‐stage disease at diagnosis as opposed to limited disease stage (PFS: HR = 1.74 [95% CI: 1.02–2.96]; OS: HR = 2.15 [95% CI: 1.15–4.02]; both P < 0.05]. Notably, undetectable EBV‐DNA levels at auto‐HCT were associated with better OS (HR = 0.29 [95% CI: 0.15–0.55]; P = 0.001) and PFS (HR = 0.46 [95% CI: 0.26–0.82]; P = 0.008), as well as reduced RI (HR = 0.43 [95% CI: 0.23–0.81]; P = 0.009) (Table 3).

In the final multivariable model, only advanced stage and EBV‐DNA viral load significantly influenced outcomes. Advanced stage at diagnosis significantly reduced OS (HR = 2.4 [95% CI: 1.05–5.51]; P = 0.04) and a trend towards reduced PFS (HR = 1.7 [95% CI: 0.88−3.28]; P = 0.11) (Figure 3). Patients with undetectable EBV‐DNA at auto‐HCT showed significantly improved OS and PFS (OS: HR = 0.28 [95% CI: 0.14–0.54]; PFS: HR = 0.36 [95% CI: 0.2–0.67]; both P = 0.001), primarily due to a lower risk of relapse (HR = 0.34 [95% CI: 0.17–0.65]; P = 0.001) (Figure S2).

## DISCUSSION

This study provides a comprehensive outcome analysis of a large cohort of NKTCL patients undergoing auto‐HCT, covering a time period during which most patients received state‐of‐the‐art first‐line therapy. There were three main findings: First, approximately 55% of the patients achieved long‐term OS and 40% long‐term PFS. Although no statistically significant difference was observed between patients undergoing auto‐HCT upfront versus later during their course of disease, this finding should be interpreted with caution. Due to the registry‐based approach, our study only included patients who ultimately could undergo auto‐HCT; this selection bias must be considered before drawing final conclusions regarding the role of timing of auto‐HCT. Second, advanced disease stage at diagnosis and the detection of EBV‐DNA in the blood at the time of transplantation were identified as poor prognostic factors, being associated with substantially decreased survival. The association of detectable EBV‐DNA levels with prognosis (3‐year OS of 38% vs. 95% for CR1 patients with detectable vs. undetectable EBV‐DNA) was particularly pronounced in patients undergoing auto‐HCT in CR after 1 treatment line. Finally, no significant differences in outcomes were observed between Asian and European patients, across PINK score groups, and for patients with prior PD‐1/PD‐L1 inhibitor treatment. Factors predicting OS and PFS were disease stage at diagnosis and undetectable EBV‐DNA in the peripheral blood at the time of auto‐HCT.

Reports evaluating auto‐HCT in larger NKTCL cohorts are scarce, and the available evidence, particularly for patients from Western countries, is primarily derived from small retrospective series. The French analysis by Walter et al. investigated outcomes of 46 NKTCL patients who underwent consolidative auto‐HCT, 26 of whom received auto‐HCT after first‐line therapy.^24^ An OS rate of 53% at 4 years was reported for auto‐HCT patients.^24^ Although specific outcomes for upfront auto‐HCT were not reported, consolidation with HCT in first CR/PR showed improved survival. In 31 patients (auto‐HCT: 26, allo‐HCT: 5) who received transplantation in first CR/PR, the 4‐year OS rate was 74% compared to 51% for those without consolidative HCT. We observed similar survival rates in autografted patients; however, our results specifically highlight the importance of remission status at transplantation, with significantly worse outcomes being observed in patients who fail to achieve a molecular remission, as indicated by detectable EBV‐DNA at auto‐HCT. Surprisingly, the 1‐year NRM rate after auto‐HCT was as high as 18% in the French study^24^—notably higher than we observed in our cohort. Still, in our cohort, NRM was higher (5.7% at 1 year) than what has been reported for other disorders after auto‐HCT,^25^ likely reflecting the toxicities of the aggressive treatment that patients with NKTCL usually endure.

A smaller retrospective analysis by a Chinese group compared outcomes of upfront auto‐HCT consolidation versus no consolidation in 40 patients with Stage III–IV NKTCL in CR1 after ASPA‐based treatment.^26^ They reported an overall 2‐year OS of 47%. In the subgroup of 20 patients who underwent upfront auto‐HCT, the 2‐year OS was 61% (vs. 28% for those without consolidation),^26^ which generally aligns with our results. Although this study suggested a benefit of auto‐HCT in CR1 compared to ASPA‐based treatment alone, missing data for EBV‐DNA response levels—as an important indicator of molecular remission—may introduce a bias and complicate cross‐study comparisons. Notably, prior studies from the pre‐ASPA‐era have also failed to demonstrate a consistent benefit of post‐induction auto‐HCT.^27^, ^28^ In contrast, our data emphasize excellent survival outcomes in patients achieving CR1 with EBV negativity, with 3‐year OS rates exceeding 90%.

The role of auto‐HCT when performed in later disease stages—such as in patients who do not respond to first‐line therapy or who experience progression—is even less clear. Although we found only numerically higher survival rates for patients receiving auto‐HCT upfront versus patients autografted after two or more treatment lines, smaller retrospective analyses, primarily conducted before the widespread adoption of ASPA‐based regimens, suggested lower survival rates for patients who underwent the procedure during a second remission.^27^, ^29^ This discrepancy might be attributed to differences in patient characteristics, treatment era, or small sample size.

To the best of our knowledge, this is the largest international study of European and Asian NKTCL patients demonstrating the prognostic significance of EBV‐DNA levels in peripheral blood before auto‐HCT. Although several studies in large cohorts of patients demonstrated that EBV‐DNA blood levels independently influence OS and PFS,^7^, ^8^, ^30^, ^31^ both in first‐line and in salvage treatment settings, our data highlight the prognostic value of EBV‐DNA before auto‐HCT. A smaller study by Lee et al. evaluated pretransplant PET‐CT and EBV‐DNA levels in a cohort of 27 patients undergoing auto‐HCT between 2009 and 2014 and similarly suggested that detectable EBV‐DNA was associated with inferior outcomes.^32^ The limited sample size of the study, however, underscores the need for validation in a larger, multinational cohort such as ours. We observed striking differences in 3‐year OS rates: approximately 27% for patients with detectable EBV‐DNA versus 78% for those without. These findings are consistent with previous reports from the Asian Lymphoma Study Group and the study by Lim et al., further supporting the predictive value of EBV‐DNA in identifying patients at risk of treatment failure.^7^, ^8^, ^32^ Given the poor results of patients with detectable EBV‐DNA before auto‐HCT regardless of their disease status at transplantation, we believe that this observation deserves further investigation and—if confirmed—alternative treatment options such as allo‐HCT should be discussed. Direct comparisons between auto‐HCT and allo‐HCT, even with retrospective data, in NKTCL are limited. Building on the results of Kanate et al. and our recent report, allo‐HCT might offer an advantage to high‐risk patients, whereas low‐risk patients may undergo auto‐HCT first.^15^, ^16^

In terms of preparative regimens for auto‐HCT, BEAM or BEAM‐like protocols were used in approximately 90% of European patients, whereas around 70% of Asian patients received TBI‐based conditioning. This finding contrasts with a previous report from Asian centers, where BEAM‐like protocols have been described as the most frequently used regimens.^29^ The frequent use of TBI‐containing regimens in this study obviously reflects the choice of conditioning by the centers contributing to this analysis. No significant differences in outcomes were observed after preparation with BEAM‐like and TBI‐based regimens; a comparison with other conditioning regimens was not possible because numbers were low. In general, although we analyzed a relatively large cohort of patients, some differences between Asian and European patients may have gone undetected because subgroups of patients with distinct characteristics were not large enough. Currently, no data are available regarding the use of ICI in the context of auto‐HCT; however, our analysis of a small cohort of 17 patients revealed no significant impact on treatment outcomes. Larger studies with extended follow‐up are required to evaluate the potential of ICI in various clinical settings and to better define the role of consolidative HCT following ICI therapy.

Our study has limitations common to all retrospective analyses. As a registry‐based investigation, we could only assess outcomes for patients who underwent auto‐HCT, excluding patients who did not proceed to transplantation due to failed salvage therapy. Additionally, restaging methods before transplant and response criteria were not standardized but depended on investigators' choice and the time of transplantation. Data on EBV‐DNA levels were available for only about two‐thirds of patients, with some variability in analysis methods across centers. Yet, we observed large differences in survival for patients with and without undetectable EBV‐DNA at auto‐HCT. This new finding certainly merits further investigation in larger cohorts of patients using comparable, precisely defined technologies.

In conclusion, our study provides new insights into the role of auto‐HCT after state‐of‐the‐art first‐line therapy and in patients with refractory and relapsed disease. For the first time, we highlight the importance of achieving CR before auto‐HCT and the particular role of EBV monitoring in predicting outcomes. Overall, our data support the use of auto‐HCT as an effective treatment strategy for eligible patients with NKTCL but also highlight the need for future research to further improve patient outcomes and explore optimized treatment strategies.

## AUTHOR CONTRIBUTIONS

**Philipp Berning**: Writing—review and editing; conceptualization; investigation; methodology; formal analysis; supervision; visualization; writing—original draft; software; data curation. **Maud Ngoya**: Methodology; software; data curation; formal analysis; writing—review and editing; investigation. **Won Seog Kim**: Writing—review and editing; investigation; conceptualization. **Evgenii Shumilov**: Writing—review and editing; investigation; conceptualization. **Depei Wu**: Writing—review and editing. **Haiwen Huang**: Data curation; writing—review and editing. **Anne Cairoli**: Data curation; writing—review and editing. **Alessandra Tucci**: Data curation; writing—review and editing. **Guillaume Dachy**: Data curation; writing—review and editing. **Romain Gounot**: Data curation; writing—review and editing. **Anne C. Wilke**: Data curation; writing—review and editing. **Christof Scheid**: Writing—review and editing; data curation. **Peter Dreger**: Data curation; writing—review and editing. **Jose Luis Lopez Lorenzo**: Writing—review and editing; data curation. **Adrian Bloor**: Writing—review and editing; data curation. **Joanna Romejko‐Jarosinska**: Writing—review and editing; data curation. **Alain Gadisseur**: Data curation; writing—review and editing. **Roland Schroers**: Writing—review and editing; data curation. **Péter Reményi**: Data curation; writing—review and editing. **Ludovic Gabellier**: Writing—review and editing; data curation. **Monica Poiani**: Writing—review and editing; data curation. **Khalid Halahleh**: Writing—review and editing; data curation. **Jacques‐Emmanuel Galimard**: Writing—review and editing; data curation; software; supervision; methodology; investigation. **Georg Lenz**: Writing—review and editing; data curation. **Anna Sureda**: Writing—review and editing; data curation. **Ali Bazarbachi**: Writing—review and editing; data curation; conceptualization; investigation. **Bertram Glass**: Conceptualization; investigation; writing—review and editing; data curation. **Norbert Schmitz**: Writing—review and editing; writing—original draft; conceptualization; methodology; supervision; formal analysis; investigation.

## CONFLICT OF INTEREST STATEMENT

The authors declare no conflicts of interest.

## FUNDING

Open Access funding enabled and organized by Projekt DEAL.

## Supporting information

Supporting Information.

## Data Availability

The data that support the findings of this study are available from the corresponding author upon reasonable request.
