# Supplementary material for: Autologous stem cell transplantation in NK/T‐cell lymphoma: Prognostic impact of EBV‐DNA in a multinational cohort—A study by the EBMT Lymphoma Working Party
Source: Hemasphere. 2025 Aug 15;9(8):e70184. doi: 10.1002/hem3.70184 (PMC12355192; doi:10.1002/hem3.70184)
Supplement: Supplementary file 1 — Supporting Information. [file HEM3-9-e70184-s001.docx]

**Supporting Information**

**Autologous stem cell transplantation in NK/T‐cell lymphoma: Prognostic impact of EBV‐DNA in a multinational cohort—A study by the EBMT Lymphoma Working Party**

**Table S1. Cooperating centers listed by patient numbers.**

| **Center** | **N** |
| --- | --- |
| Samsung Medical center, Seoul, South Korea | 43 |
| First Affiliated Hospital of Soochow University, Suzhou, China | 13 |
| CHUV Centre Hospitalier Universitaire Vaudois, Lausanne, Switzerland | 5 |
| Spedali Civili – Brescia, Brescia, Italy | 4 |
| Cliniques Universitaires St. Luc, Brussels, Belgium | 4 |
| Hopital Henri Mondor, Creteil, France | 4 |
| University Hospital Frankfurt - Goethe University, Frankfurt Main, Germany | 4 |
| University of Cologne, Cologne, Germany | 4 |
| University of Heidelberg, Heidelberg, Germany | 3 |
| Fundación Jiménez Díaz, Madrid, Spain | 3 |
| Christie Hospital Manchester, Manchester, United Kingdom | 3 |
| Maria Sklodowska-Curie Institute - Oncology Centre, Warsaw, Poland | 3 |
| Antwerp University Hospital (UZA), Antwerp E, Belgium | 2 |
| ZSIS Universitaetsklinikum Knappschaftskrankenhaus Bochum, Bochum, Germany | 2 |
| Dél-pesti Centrumkórház, Budapest, Hungary | 2 |
| CHU Lapeyronie, Montpellier, France | 2 |
| Azienda Sanitaria Universitaria Giuliano Isontina, Trieste, Italy | 2 |
| King Hussein Cancer Centre Adult BMT Program, Amman, Jordan | 2 |
| Angers CHRU, Angers, France | 1 |
| Gazi University, Ankara, Turkey | 1 |
| University Hospital Bern, Bern, Switzerland | 1 |
| Jules Bordet Institute, Brussels, Belgium | 1 |
| Caen University Hospital Hematology Department, Caen, France | 1 |
| University Medical Center Groningen, Groningen, Netherlands | 1 |
| University Medical Center Hamburg-Eppendorf, Hamburg, Germany | 1 |
| University Medical Center Schleswig-Holstein (UKSH), Kiel, Germany | 1 |
| Manchester Royal Infirmary, Manchester, United Kingdom | 1 |
| San Raffaele Hospital, Milan, Italy | 1 |
| University Hospital Muenster, Muenster, Germany | 1 |
| Cardarelli Hospital, Naples, Italy | 1 |
| University Hospital Ostrava, Ostrava, Czech Republic | 1 |
| King Faisal Specialist Hospital & Research Centre, Riyadh, Saudi Arabia | 1 |
| Erasmus Medical Center, Rotterdam, Netherlands | 1 |
| Saint-Etienne University Hospital, Saint-Étienne, France | 1 |
| University Hospital of Salamanca, Salamanca, Spain | 1 |
| Hospital Universitario Marqués de Valdecilla, Santander, Spain | 1 |
| St. Marien Hospital, Siegen, Germany | 1 |
| Diakonie-Klinikum Stuttgart, Stuttgart, Germany | 1 |
| Robert Bosch Hospital, Stuttgart, Germany | 1 |
| University Hospital Bretonneau, Tours, France | 1 |
| Turku University Hospital, Turku, Finland | 1 |
| Gustave Roussy Cancer Campus, Villejuif, France | 1 |
| St. Augustinus Hospital, Wilrijk, Belgium | 1 |

**Table S2. Disease Status at auto-HCT and number of prior treatment lines by stage at diagnosis.**

| **Stage at Diagnosis** | **Remission at auto-HCT** | | | | **Number of treatment line** | |
| --- | --- | --- | --- | --- | --- | --- |
|  | CR | PR | PD / Relapse | SD | 1 prior line | ≥2 prior lines |
| **I/II (n = 42)** | 26 (61.9%) | 12 (28.6%) | 3 (7.1%) | 1 (2.4%) | 12 (28.6%) | 28 (66.7%) |
| **III/IV (n = 78)** | 44 (56.4%) | 22 (28.2%) | 8 (10.3%) | 4 (5.1%) | 53 (67.9%) | 26 (33.3%) |

Abbreviations: CR=complete remission; PR=partial remission; PD=progressive disease; SD=stable disease.

**Table S3. Clinical and transplantation characteristics by region.**

| **Variable** | **Asia**  **N = 56 (%)** | **Europe**  **N = 74 (%)** | ***P-value*** |
| --- | --- | --- | --- |
| Age at transplantation, median, range (years) | 50.2 (23-69.4) | 51.9 (23.1-72.8) | 0.236 |
| Diagnosis – auto-HCT median, range | 7.6 (3.4-156.8) | 7.4 (2.8-174.9) | 0.875 |
| Male | 36 (64.3) | 51 (68.9) | 0.578 |
| ECOG at auto-HCT |  |  |  |
| 0-1 | 25 (100) | 33 (91.6) |  |
| ≥ 2 | 0 | 3 (8.3) |  |
| Unknown | 31 | 38 |  |
| Ann-Arbor stage at diagnosis |  |  | 0.839 |
| Localized (I-II) | 20 (35.7) | 22 (33.8) |  |
| Advanced (III-IV) | 36 (64.3) | 43 (66.2) |  |
| Unknown | 0 | 9 |  |
| PINK score |  |  | <0.001 |
| High | 7 (12.5) | 31 (47.7) |  |
| Low/Intermediate | 49 (87.5) | 34 (52.3) |  |
| Unknown | 0 | 9 |  |
| Disease type / manifestations |  |  | 0.254 |
| Nasal-type | 27 (62.8) | 35 (48.6) |  |
| Extranasal | 16 (37.2) | 37 (51.4) |  |
| Unknown | 13 | 2 |  |
| First-line therapy |  |  |  |
| Anthracycline-based | 2 (3.6) | 14 (18.9) |  |
| Asparaginase-based | 37 (66.1) | 48 (64.9) |  |
| Platinum-based (DeVIC/VIPD/ICE) | 1 (1.8) | 10 (13.5) |  |
| Radiotherapy only | 13 (23.2) | 1 (1.4) |  |
| Other | 3 (5.4) | 1 (1.4) |  |
| Number of prior therapies |  |  | 0.021 |
| 1 | 29 (51.8) | 39 (54.2) |  |
| 2 | 13 (23.2) | 27 (37.5) |  |
| 3 or more | 14 (25) | 6 (8.3) |  |
| Unknown | 0 | 2 |  |
| Asparaginase-containing therapy (any time before HCT) | 47 (83.9) | 50 (75.8) | 0.265 |
| PD-1/PD-L1 inhibitor treatment |  |  | <0.001 |
| No PD-1/PD-L1 inhibitor | 42 (75) | 66 (95.7) |  |
| Before HCT | 14 (25) | 3 (4.3) |  |
| Unknown | 0 | 5 |  |
| Status at transplantation |  |  | 0.104 |
| CR | 27 (49.1) | 50 (67.6) |  |
| PR | 19 (34.5) | 17 (23) |  |
| SD/PD | 9 (16.4) | 7 (9.5) |  |
| Unknown | 1 | 0 |  |
| Conditioning regimen |  |  | <0.0001 |
| BEAM | 1 (2.2) | 45 (61.6) |  |
| BEAM-like | 12 (26.7) | 21 (28.8) |  |
| TBI based | 32 (71.1) | 5 (6.8) |  |
| TBI + BEAM / BEAM-like | 0 | 2 (2.7) |  |
| Unknown | 11 | 1 |  |
| Subsequent allo-HCT | 2 (4.5) | 11 (20) | 0.023 |
| Abbreviations: Auto-HCT=autologous hematopoietic stem cell transplantation; ECOG=Eastern cooperative oncology group; PINK=prognostic index for NK/T-cell lymphoma; DeVIC=dexamethasone, etoposide, ifosfamide, carboplatin; VIPD=etoposide, ifosfamide, cisplatin, dexamethasone; CR=complete remission; PR=partial remission; SD=stable disease; PD=progressive disease; EBV=Epstein-Barr virus; TBI=total body irradiation; allo-HCT=allogeneic hematopoietic stem cell transplantation. | | | |

**Table S4.** **Univariate analysis of post-transplantation outcomes for selected subgroups.**

| **Variable** | **Outcome** | **3-year probability [95% CI]** | | ***P*** |
| --- | --- | --- | --- | --- |
| **Disease type at diagnosis** | | **Nasal-type** | **Extranasal manifestations** |  |
|  | OS | 61.7%[47.7-72.9] | 70.1%[55.2-80.9] | 0.89 |
|  | PFS | 50.6%[36.7-62.9] | 54.7%[39.7-67.4] | 0.70 |
|  | RI | 46%[32.3-58.7] | 39.2%[25.4-52.7] | 0.53 |
|  | NRM | 3.4%[0.6-10.6] | 6.1%[1.6-15.3] | 0.63 |
| **PD-1/PD-L1 inhibitor before auto-HCT** | | **No** | **Yes** |  |
|  | OS | 63.7%[53.5-72.3] | 63%[29.3-84.1] | 0.73 |
|  | PFS | 50%[39.7-59.5] | 41.7%[16.4-65.4] | 0.27 |
|  | RI | 43.2%[33.1-52.9] | 58.3%[26.6-80.3] | 0.11 |
|  | NRM | 6.8%[3-12.7] | 0% | 0.31 |
| **EBV DNA in CR1 patients at auto-HCT** | | **Detectable** | **Not Detectable** |  |
|  | OS | 37.5% [8.7-67.4] | 95.0% [69.5-99.3] | 0.01 |
|  | PFS | 37.5% [8.7-67.4] | 58.4% [24.4-81.5] | 0.39 |
|  | RI | 62.5% [18.5-87.7] | 36.4% [8.5-66.0] | 0.27 |
|  | NRM | 0% | 5.3% [0.3-22.0] | 0.53 |
| **Advanced stage (III/IV) and timing of auto-HCT** | | **Upfront (1 line)** | **2 or more lines** |  |
|  | OS | 64.1% [49.1-75.8] | 40.6% [21.6-58.8] | 0.21 |
|  | PFS | 49.9% [34.6-63.3] | 26.7% [11.1-45.2] | 0.29 |
|  | RI | 44.1% [29.1-58.0] | 61.3% [37.7-78.3] | 0.41 |
|  | NRM | 6.1% [1.5-15.2] | 12.0% [2.9-28.2] | 0.91 |
| **No anthracycline-based pre-treatment by timing of auto-HCT** | | **Upfront (1 line)** | **2 or more lines** |  |
|  | OS | 63.9% [49.8–75] | 57.1% [41.7–69.9] | 0.93 |
|  | PFS | 50.4% [36.7–62.6] | 42.2% [27.5–56.1] | 0.87 |
|  | RI | 44.6% [31.1–57.2] | 51.3% [35.4–65.1] | 0.92 |
|  | NRM | 5.0% [1.3–12.6] | 6.5% [1.7–16.2] | 0.84 |
| **Conditioning treatment** | | **BEAM / BEAM-like** | **TBI-based** |  |
|  | OS | 71.8% [60–80.6] | 52.5% [35.1–67.4] | 0.18 |
|  | PFS | 52.5% [40–63.6] | 38.8% [23–54.4] | 0.51 |
|  | RI | 43.3% [31.2–54.8] | 53.1% [34.9–68.2] | 0.56 |
|  | NRM | 4.2% [1.1–10.7] | 8.1% [2–19.8] | 0.98 |
|  |  |  |  |  |
| Abbreviations: CI=confidence interval; PFS=progression-free survival; OS=overall survival; RI=cumulative incidence of relapse; NRM=cumulative incidence of non-relapse mortality; auto-HCT=autologous hematopoietic stem cell transplantation; EBV=Epstein-Barr Virus; PINK=prognostic index for NK/T-cell lymphoma; CR=complete remission; BEAM=carmustine, etoposide, cytarabine, melphalan; TBI=total body irradiation; | | | | |

**Figure S1. Overall survival of NKTCL patients by PINK score groups and remission status.**

Kaplan-Meier estimates for overall survival of indicated subgroups. (A) PINK score groups low/intermediate vs. high. (B) Patients with advanced disease stage (III/IV) by timing of auto-HCT. (C) CR after 1 line of treatment (CR1) vs. CR after 2 or more treatment lines. PINK= Prognostic Index for Natural Killer/T-cell lymphoma; CR=complete remission; PR=partial remission; SD/PD= stable disease/progressive disease; CR1=complete remission after 1 treatment line; CR2=complete remission after 2 or more treatment lines.

**Figure S2. Forest plots of multivariable analysis of prognostic factors for RI and NRM.**

The hazard ratios (HR) and corresponding 95% confidence intervals (95% CI) were obtained from multivariable regression using the Cox proportional hazards model. These results pertain to the independent variables evaluated for predicting post-auto-HCT outcomes in all NKTCL patients. RI=Relapse incidence; NRM=Non-relapse mortality; Ref.=Reference group; PINK=Prognostic Index for NK/T-cell lymphoma; CR=complete remission; PR=partial remission; SD/PD=stable disease/progressive disease.

**Figure S1. Overall survival of NKTCL patients by PINK score groups and remission status.**

**
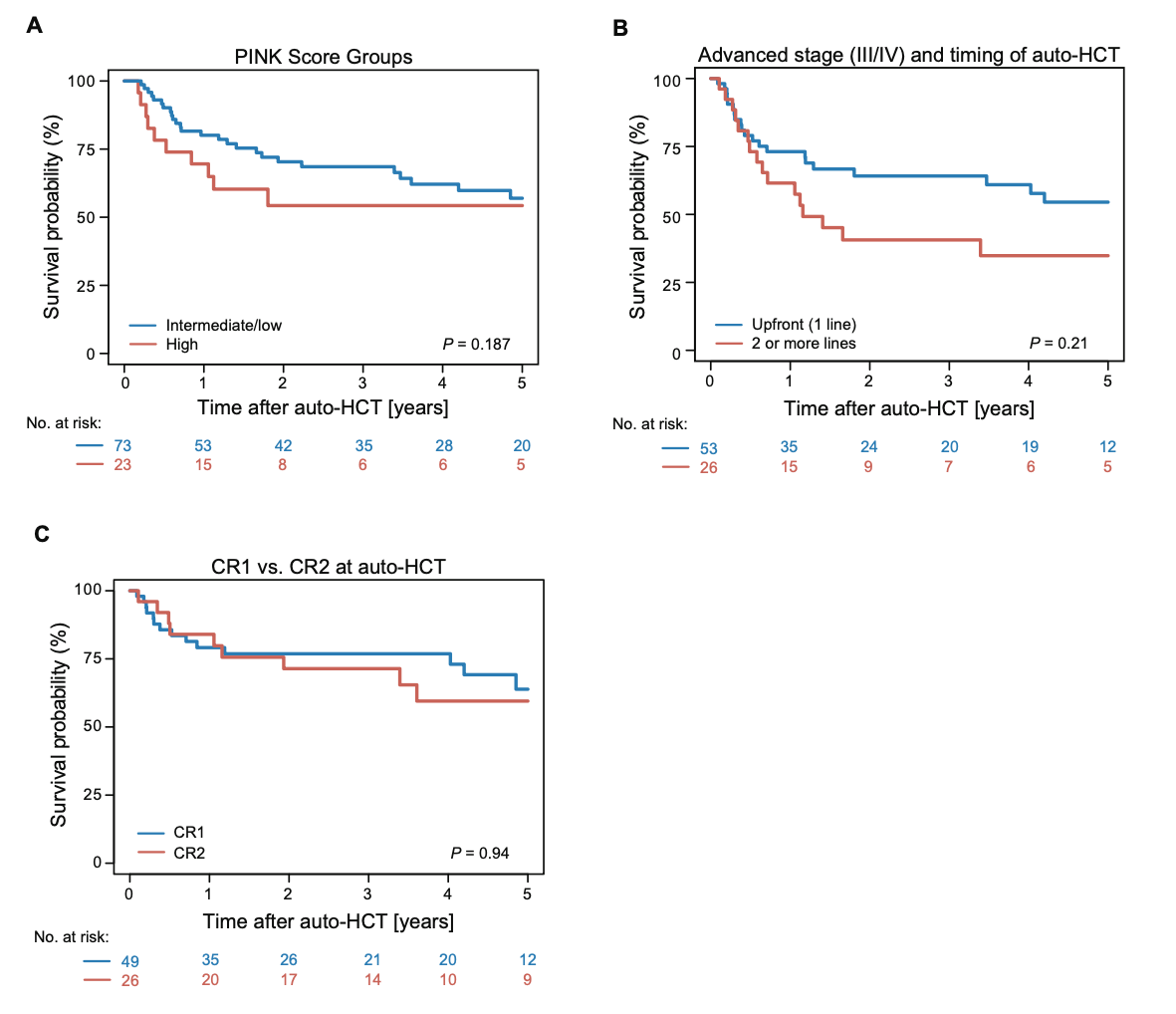
**

**Figure S2. Forest plots of multivariable analysis of prognostic factors for RI and NRM.**

**
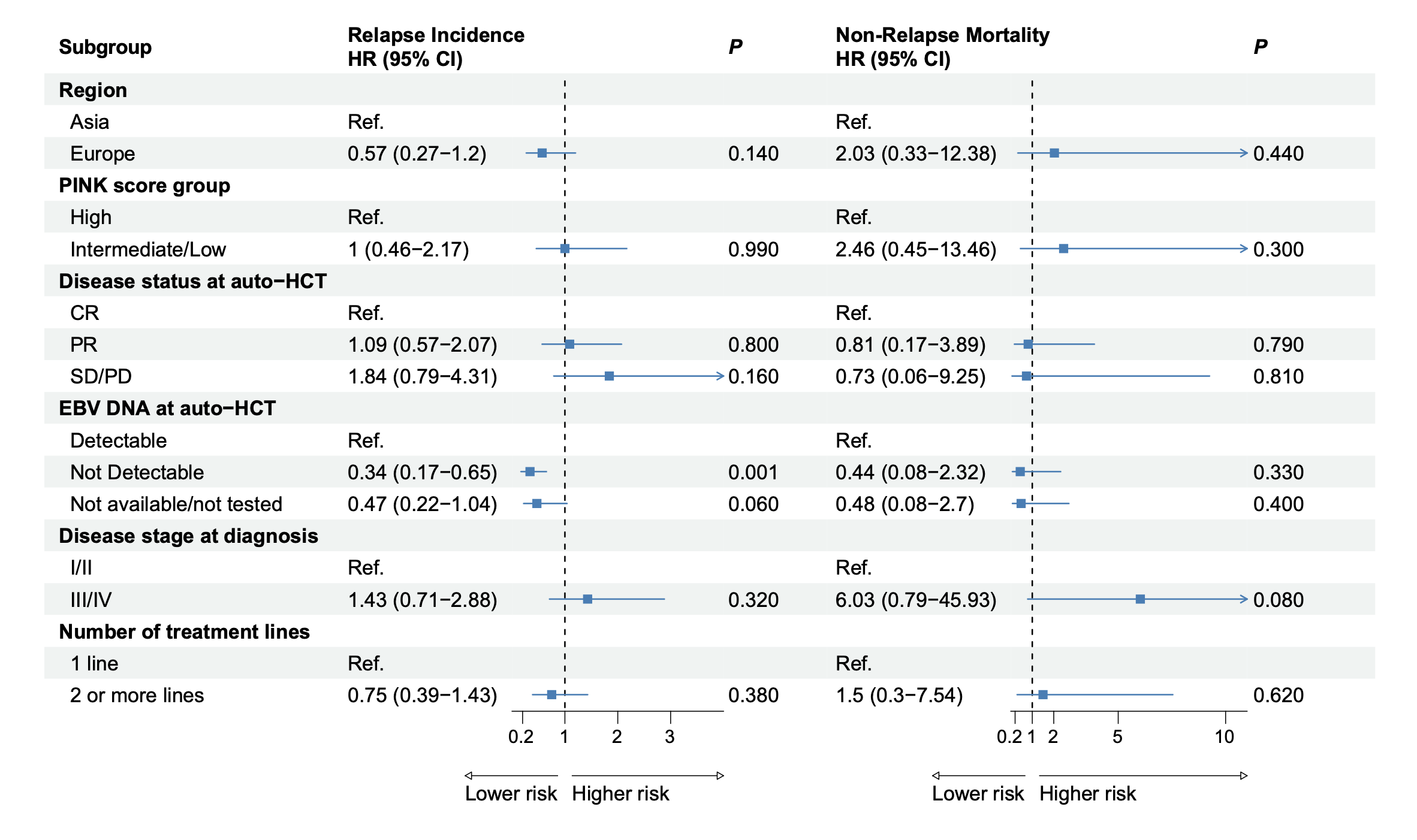
**
